# Antibacterial Cotton Fabric Functionalized with Copper Oxide Nanoparticles

**DOI:** 10.3390/molecules25245802

**Published:** 2020-12-09

**Authors:** Luz E. Román, Enrique D. Gomez, José L. Solís, Mónica M. Gómez

**Affiliations:** 1 Faculty of Science, Universidad Nacional de Ingeniería, Av. Túpac Amaru 210, Lima 15333, Peru; luz.esmeralda.roman@uni.pe (L.E.R.); jsolis@uni.edu.pe (J.L.S.); 2 Department of Chemical Engineering, The Pennsylvania State University, University Park, PA 16802, USA; edg12@psu.edu; 3 Department of Materials Science and Engineering, Materials Research Institute, The Pennsylvania State University, University Park, PA 16802, USA

**Keywords:** antibacterial, copper oxide, nanoparticles, functionalization, textile

## Abstract

Textiles functionalized with cupric oxide (CuO) nanoparticles have become a promising option to prevent the spread of diseases due to their antimicrobial properties, which strongly depend on the structure and morphology of the nanoparticles and the method used for the functionalization process. This article presents a review of work focused on textiles functionalized with CuO nanoparticles, which were classified into two groups, namely, in situ and ex situ. Moreover, the analyzed bacterial strains, the resistance of the antimicrobial properties of textiles to washing processes, and their cytotoxicity were identified. Finally, the possible antimicrobial mechanisms that could develop in Gram-positive and Gram-negative bacteria were described.

## 1. Introduction

Infectious diseases are caused by pathogenic microorganisms such as bacteria, viruses, parasites, and fungi [1]. Managing infectious diseases is of great interest for the sake of public health, and each year there is a greater demand for studies due their growing impact worldwide, both in developed and developing countries [2,3]. Infectious diseases have a clear impact on the community and health care facilities. In addition, infectious agents continue to emerge and reemerge with new resistance worldwide [4]. A good example is the coronavirus disease 2019 (COVID-19), which is caused by severe acute respiratory syndrome coronavirus 2 (SARS-CoV-2) [5]. This novel coronavirus is genetically related to the Middle Eastern Respiratory Syndrome virus (MERS-CoV) and the Severe Acute Respiratory Syndrome virus (SARS-CoV) [6]. Coronavirus disease 2019 attacks not only the respiratory system but also other organs, such as the liver and the heart [7,8] and, due to its transmission directly or indirectly from one person to another, it is causing high mortality and morbidity rates [6]. Globally, as of 12th October 2020, there were a total of 37,423,660 confirmed cases, including 1,074,817 deaths [9]. This novel virus or other pathogens can disseminate through well-defined routes via hands and from contaminated common-touch surfaces to new surfaces and/or other hosts [4,10]. These common-touch surfaces in interior spaces can be doorknobs, toilet flush handles, faucet handles, digital devices, and light switches, among others [4].

Just like COVID-19, Healthcare-Associated Infections (HAIs) can be disseminated through the same routes described above (hands or contaminated surfaces) but are located in hospital environments [11,12]. HAIs are infections acquired during the hospital stay of patients, and they appear 48 h after their admission to the health center or within 30 days after their discharge [13]. Furthermore, HAIs are a serious public health problem around the world, and they are associated with additional treatment costs, complications, reduction in quality of life, and mortality [14,15]; however, the current number of patients who contract them is unknown due to the difficulty in gathering reliable data in countries that lack surveillance systems for this type of infection [16]. In 2013, the European Center for Disease Prevention and Control estimated a total of 4.2 million HAIs in European long-term care centers, which represented more than 116,000 cases per day [15,17]. Conversely, this type of infections can be prevented and controlled, and hand hygiene, hygiene and sanitation and the correct use of personal protective equipment (PPE) by health-care workers are some of the essential elements to combat them [13,18]. The proper use of gloves, hats, isolation clothing, protective clothing, and other PPEs is an important prevention practice to limit the spread of HAIs in health centers and to protect patients and health-care workers [14,18]. Therefore, protective clothing, such as uniforms of health-care workers made with functionalized materials with antimicrobial finishing agents, could be an alternative to reduce and prevent COVID-19 and HAIs [15,19], given that the development of a vaccine involves months of research and, worse still, its protection can decrease in a short time, as in the case of influenza vaccines that do not exactly match the virus because it evolves rapidly [20]. Copper-based antimicrobial agents could be employed to develop these protective garments.

Copper is a metal that belongs to the eleventh group of the periodic table. There are two copper compound series depending on its valence state: the first series is the cuprous compounds that come from cuprous oxide, Cu_2_O (red), and have Cu^+1^ ion; the second series is the cupric compounds that come from cupric oxide, CuO (black), and have Cu^+2^ ion [21,22]. In the case of CuO, it is a p-type semiconductor with a band gap value of approximately 1.4 eV [21,23]. It has a monoclinic crystal structure, whose space group is *C*2/*c*, and its lattice parameters are *a* = 4.6837 Å, *b* = 3.4226 Å, *c* = 5.1288 Å, and *β* = 99.54° [23,24]. At the nanoscale, CuO is characterized by being stable and having a long shelf life [25] and exhibits remarkable applications in catalysis [26], high-temperature superconductors [27], solar cells [28,29], chemical and gas sensors [30], and lithium ion batteries [31], among others. Apart from those, they can be used also in medicine as antibacterial [25,32,33], antiviral [34,35], and antifungal [36,37,38] treatments. Contrarily, the application of cupric oxide (CuO) nanoparticles (NPs) on textile materials can be conducted by different methods that will be detailed in the next session. This review aims to highlight research on antimicrobial textiles functionalized with CuO NPs while commenting on the divergent methodologies employed in the development of this type of fabric, analysis of antimicrobial activity, wash durability test, some uses, a brief description of cytotoxicity, and future prospects.

## 2. Textile Functionalization Methodologies

Functionalization with nanoparticles of materials made with cotton fibers or other types of fibers can be classified into two methods: in situ (one-step process) and ex situ (two-step process) [39,40,41].

**Figure 1:**
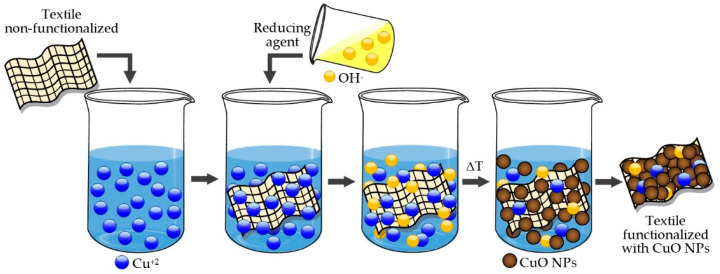
Scheme of textile functionalization by the in situ method.

The in situ method consists of synthesizing nanoparticles in the presence of textile materials, as shown in Figure 1. For instance, Perelshtein and colleagues [42] irradiated a copper acetate solution with ultrasound, over which a 100% cotton bandage was immersed, and then an ammonium-based reducing agent was added to synthesize CuO onto the cotton textile. By X-ray diffraction (XRD), the authors confirmed that the NPs present in the textile corresponded to the monoclinic crystalline phase of CuO. This method of functionalization by ultrasound not only allows obtaining textiles with antimicrobial properties against *Escherichia coli (E. coli)* and *Staphylococus aureus (S. aureus)* bacteria but also provides good resistance to washing cycles. As indicated in the article, CuO content in textiles, which was determined by volumetric titration with EDTA and controlled by inductive coupled plasma (ICP), had no reduction after 20 washing cycles at 40 °C. The leaching of copper ions (Cu^+2^) was also examined by ICP, and for this, a small sample of textiles functionalized with 1.4% CuO (*wt%*) was immersed in an aqueous solution of sodium chloride (NaCl) overnight at 37 °C. The concentration of leached Cu^+2^ ions was 0.15 ppm, and this amount represented only 1% of the total functionalized copper. Furthermore, functionalization was not removed in the pH range between 6 and 9. A description of the chemical products, contact time between textiles, pH of solutions and technical standards that were employed would have been useful, to ascertain the possibility of working with more acidic pHs and analyzing their behavior. A sonochemical method was also utilized for synthetic fibers or the mixture of synthetic and natural fibers [43,44,45,46,47].

Another method for in situ functionalization is by dip-coating. In this method, some authors have performed a pretreatment of textiles before functionalization. For instance, Markovic et al. [48] washed cotton fabrics with a nonionic detergent solution and then dipped them in oxalic acid solutions of different concentrations to modify their surface and create carboxyl groups that improve the anchoring of Cu^+2^ ions. Nabil et al. [49] also treated cotton fibers with an aqueous solution composed of hydrogen peroxide and ammonium hydroxide to hydroxylate the cotton, that is, to introduce hydroxyl groups that would bind or be replaced with other chemical products that would be added after the functionalization with CuO. In the case of the group led by Rezaie, they previously washed a wool sample with a nonionic detergent solution to remove impurities [50]. Subsequently, to synthesize CuO NPs in the presence of textile materials, the authors immersed and stirred the samples with previous treatments in solutions of copper precursor salts, such as copper sulfate or acetate, and then treated them with alkaline solutions from an industrial source (sodium hydroxide or sodium borohydride) or solutions obtained from natural sources, as they did in Rezaie’s research, who used the supernatant produced after dissolving the ashes of burnt leaves and the stems of the *Seidlitzia rosmarinus* plant in water. Markovic et al. provided a clear example of the surface treatment of cotton to establish carboxyl groups from oxalic acid that uptake of Cu^+2^ ions in the in situ functionalization process with CuO NPs. The research also indicated that cotton textiles with a greater amount of oxalic acid uptake more copper ions, which could be visualized in electron micrographs, and correlated with excellent microbiological results against Gram-positive and Gram-negative bacteria. Conversely, the uptake of Cu^+2^ ions from cotton without previous treatment was not reported, and this information would have been useful to verify and compare the influence of oxalic acid on the uptake of ions before and after its application. An important aspect is that this study determined the oxidation state of the copper present in textile samples, and it also reported that the cotton treated with the highest amount of oxalic acid presented a mixture of CuO and Cu_2_O oxides, with CuO being the most abundant. In examining the methodology and results of the study by Nabil et al. it was apparent that they obtained cotton fabrics with CuO nanoparticles and grafting of organo-silane and diethanolamine by using the in situ method. One of their main results was that the samples functionalized with CuO had a high catalytic activity to reduce methylene-blue and 4-nitrophenol that can be present in wastewater as contaminants, despite the fact that the samples presented a fairly heterogeneous distribution of nanoparticles on their surface. These same samples did not have such good antimicrobial activity against *Staphylococcus epidermidis (S. epidermidis)* bacteria, with an inhibition zone value of between 0 and 3 mm compared with the *E. coli* strain with an inhibition zone value of between 4 and 6 mm.

**Figure 2:**
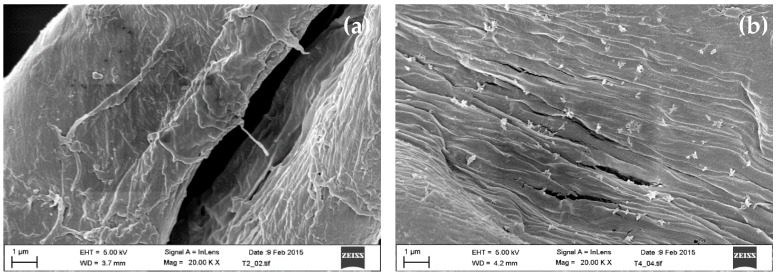
FESEM micrographs of cotton fabrics: (**a**) non-functionalized and (**b**) In Situ functionalized with CuO NPs.

In a study developed by our research group, the cotton fabrics were functionalized with CuO NPs by employing an exhaust dyeing method. The copper acetate and sodium hydroxide were also used for functionalization. The microbiological results validated that the functionalized fabrics inhibited the growth of *E. coli* bacteria; apart from this, it was proposed that a possible route for interaction mechanism between CuO and cotton fiber is through the OH-groups of cellulose, that is, a complex-fiber bonding could have become a seed for the CuO NP [51]. In this study and by scanning electron microscopy, the morphology of cotton fiber before and after the functionalization process can be appreciated (Figure 2). For the non-functionalized fabric displayed in Figure 2a, a clean fiber is seen, that is, one without the presence of any particle on its surface. In the case of the functionalized fabric (Figure 2b), there are randomly distributed particles on the cotton fiber, and it was determined that these particles corresponded to CuO by using XRD.

Apart from the functionalization the in situ method mentioned before, there are others shown in Table 1, and additional information is provided, such as the following: nature of textile fibers; shape, size and structure of nanoparticles, additional treatments before or during the functionalization process; functionality; washing tests; antimicrobial tests; antimicrobial activity against different Gram-positive and Gram-negative bacterial strains.

**Figure 3:**
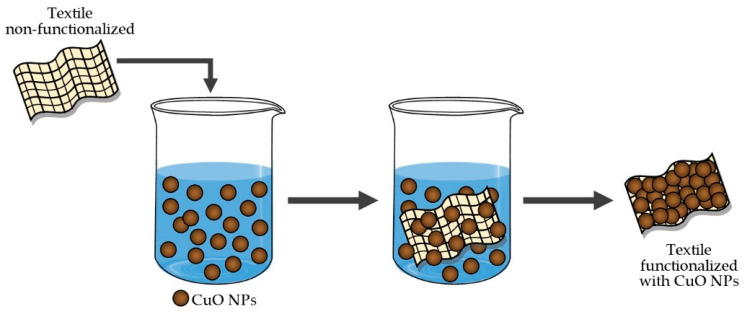
Scheme of the second step for textile functionalization by the ex situ method.

In the case of the ex situ method, there are two steps. The first step consists of synthesized nanoparticles, and then, in the second step, these nanoparticles are applied to textile materials [39,40]. Figure 3 shows a scheme of the second step of the ex situ method.

In the study developed by Thampi et al. [69], the synthesis of CuO NPs by the chemical precipitation method and its subsequent impregnation on the surface of woven fabrics and non-woven fabrics are explained. A mixture of solutions containing copper nitrate and polyethylene glycol was employed to obtain CuO NPs powder (first step of the ex situ method). These nanoparticles were employed to prepare colloids where the fabric was immersed, stirred, and then dried, and this last part corresponds to the second step of the ex situ method. Apart from this, a polymeric matrix composed of polyaniline was used to immobilize CuO to reduce the release of Cu^+2^ ions from fabrics to the surrounding environment. A certain amount of synthesized CuO was added to a dissolution aniline to obtain that immobilization, and its application onto fabrics was similar to the impregnation process described for the pure CuO. This study is a clear example of the two steps of the ex situ functionalization method. Likewise, an outstanding point to appreciate was the identification of living or dead cells on a bacterial population through fluorescein isothiocyanate and propidium iodide dual stains. The fabrics functionalized with CuO NPs were put in contact with a bacteria culture. After a certain time, the aliquots were taken from this culture, and the dual staining was added. By epi-fluorescence microscopy, it was visualized that bacteria before contact with copper ions released from fabrics had a green staining that represents living cells. After 6 h of contact, the bacterial strain presented a slight reddish coloration, but after 10 h of contact, the red color was intensified, confirming the bacterial death. In addition, these results can also be correlated with the information displayed by scanning electron microscopy, where the dead bacteria showed shrinkage after being in contact with the CuO functional textile. Another example of functionalization by the ex situ method was developed by Vasantharaj et al. [70]. A copper sulfate solution and an aqueous extract of *Ruellia tuberosa* leaves that reduce the copper toxicity were employed for the green synthesis of CuO NPs. Thereafter, these nanoparticles were used to coat cotton fabrics and impart bactericidal properties to them. The authors by means of UV-visible spectra and vibrational characterization as Fourier transform infrared spectroscopy confirmed that the synthesized nanoparticles were composed of CuO. Contrarily, although the reported results exhibited a good antibacterial activity for the CuO NPs evaluated in three different concentrations (25, 50, and 75 µg/mL) and for the fabrics coated with the nanoparticles, they did not indicate the amount of CuO that was applied on the textile material during the functionalization process. This information would have been useful to compare and to observe differences between the halo sizes originated by the powder nanoparticles and the functionalized textile, both with concentrations of known CuO NPs.

Table 2 mentions a summary of another synthesis procedure and the functionalization of textile materials with CuO NPs by the ex situ method.

## 3. Antimicrobial Activity of Textiles Functionalized with CuO Nanoparticles

Bacteria are prokaryotic cells whose length is between 0.2 and more than 10 µm, and their width is approximately from 0.2 to 1.5 µm [79,80]. More than 5000 species of bacteria that can be found in environments where there is liquid water are distinguished and recognized by their structural and biochemical characteristics. Additionally, they present divergent shapes, such as cocci, rods, spirals, and cubes [79,81]. Bacteria have an internal structure composed of nuclear materials or prokaryotic DNA, ribosomes, and several inclusion bodies, and they also have an external structure where cytoplasmic membrane, cell wall, flagella, pili, and other components are found [79,82]. The cytoplasmic or cell membrane is located between the cytoplasm and cell wall. This membrane has a thickness of from 7 to 8 nm and is composed of a semipermeable lipid bilayer, which generally comprises phospholipids [79,81]. Likewise, in this bilayer, there are different types of proteins that can pass through it completely or be adhered to its exterior or interior; approximately 70% of the weight of the membrane is composed of proteins [81,82]. The cytoplasmic membrane functions as a barrier that separates the interior from the exterior of the bacterial cell and allows the passive diffusion of solutes and water. It also participates in active molecular transport through protein pumps, which maintain and regulate the internal ionic concentration and the osmotic pressure of the bacterial cell [79].

**Figure 4:**
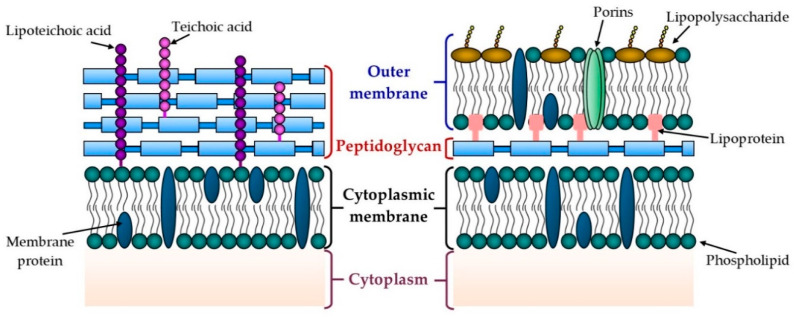
Schematic representation of Gram-positive (**left**) and Gram-negative (**right**) bacteria.

Another component of the bacterial external structure is the cell wall, which has a variety of functions, including protecting the integrity of the bacterial cell (i.e., maintaining cell shape), supporting the internal osmotic pressures of 5–20 atmospheres, allowing the interaction of microorganisms with a variable environment and the passage of macromolecules from the outside to the cytoplasmic membrane and then to the cytoplasm, and preserving the cytoplasmic membrane from the possible disruption caused by chemical products [79,80,81]. With the staining method development by Christian Gram in 1884, it was possible to distinguish the differences in the cell wall structure of bacteria, and this allowed them to be classified into two groups: Gram-positive and Gram-negative bacteria [80,83,84]. Figure 4 presents the general scheme of the structure of each of these bacteria.

Gram-positive bacteria have a cell wall composed of a 30–100-nm-thick peptidoglycan layer and teichoic acids [83]. The peptidoglycan or murein is constructed from linear glycans that are cross-linked by short peptides. Glycans comprise disaccharides composed of N-acetylglucosamine and N-acetylmuramic acids that are connected through β(1,4) glycosidic bonds [80,83,85]. Teichoic acids are anionic glycopolymers, which give a negative charge to the Gram-positive cell wall [80]. There are two classes: the teichoic acids covalently attached to the N-acetylmuramic acid of the peptidoglycan, and the lipoteichoic acids that are anchored to the cytoplasmic membrane [83].

In the case of Gram-negative bacteria, their cell wall consists of a thin layer of 2–7-nm-thick peptidoglycan and an outer membrane, and the binding between these two is through lipoproteins [81,83]. Conversely, the outer membrane is made up of an asymmetrical lipid bilayer whose inner side is similar to a cytoplasmic membrane, but its outer side is different due to the presence of lipopolysaccharides [79,81]. In this outer membrane, there are proteins that tend to be organized into trimers called “porins,” and these porins have a hydrophilic channel that permits the entry of low-molecular-weight compounds from the external environment into the bacterial cell [79,80,81].

**Figure 5:**
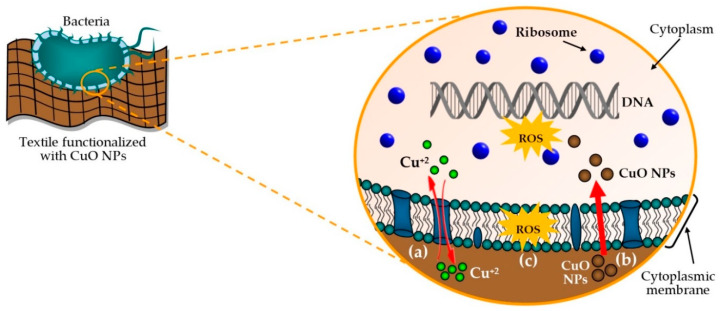
Diagram presentation of the mechanisms of the antimicrobial activity of textile materials functionalized with CuO NPs: (a) release of copper ions, (b) contact of CuO NPs with bacteria, and (c) reactive oxygen species.

The mechanism of the antimicrobial activity of functionalized textile materials with CuO NPs against Gram-positive and Gram-negative bacteria is not yet fully understood. That is why within some published studies, it is proposed that mechanism starts with the adsorption of bacteria on the textile surface [57,86]. In this condition, the antibacterial activity can be attributed to three mechanisms, namely, the release of copper ions (Cu^+2^), the direct contact of CuO NPs with bacteria, and the production of reactive oxygen species (ROS) [40,87,88]. Figure 5 presents the diagram of the antimicrobial activity of CuO NPs by the three mechanisms mentioned above.

In the case of the release of copper ions (Cu^+2^), this can be caused by the dissolution of the CuO NPs in the presence of water and oxygen [61,86,89,90]. The slight solubility of CuO could be related to its low-solubility product constant (K_sp_~10^−20^ at 25 °C), which indicates that in an aqueous solution, there is a minimum concentration of Cu^+2^ [52,66,73]. Apart from that, the water necessary to generate the dissolution of CuO could come from the medium surrounding the bacteria [57,86]. It should be considered that polar materials like cotton and non-polar, such as polyethylene or polypropylene, allow the diffusion of water molecules through their polymeric matrices, and this diffusion increases with the polarity of materials [40,86]. Thus, the water molecules emerging from the bacterial medium when coming into contact with the textile material could diffuse and interact with CuO NPs that are on the surface or embeded within the material’s polymeric matrix to later generate its dissolution, and consequently the release of Cu^+2^ ions [90]. The anionic characteristic of the bacterial cell envelope allows it to interact through electrostatic forces with extracellular molecules and metal ions [83,91]. Hence, due to this characteristic, the copper ions released could interact electrostatically with the components of the cell envelope (cell wall and cytoplasmic membrane) [87]. This interaction would cause the denaturation of proteins in the bacterial membrane and would produce the bacteriolytic effect, that is, the disintegration of the bacteria [40]. Furthermore, according to Markovic et al. and Shankar et al. copper ions bind to the cytoplasmic membrane and to the proteins responsible for metabolic processes [61,90]. In this situation, the ions can enter the cytoplasm through integral proteins or through the cytoplasmic membrane itself [61,91]. Once inside the cytoplasm, the copper ions can interact with the DNA of bacteria and become involved in the crosslinking of nucleic acid chains, generating a disorder in helical structures and thus preventing bacterial reproduction and cell death [40,87,89,90]. Figure 5(a) depicts the possible route of the entry of copper ions into the cytoplasm. To verify that the mechanism of the antibacterial activity of a material functionalized with CuO NPs is due to Cu^+2^ ions, Yoosefi et al. [66] compared the results of the microbiological analysis of the bacterial solutions obtained after being in contact: (a) with a cellulose paper sample (1 cm^2^) functionalized with CuO NPs, and (b) with leached copper ions that were produced after shaking a cellulose paper sample (1 cm^2^) functionalized with CuO NPs in a culture medium without bacteria. The reported results verified that the leached solution had the same antimicrobial activity as the solution in direct contact with the functionalized cellulose paper with nanoparticles. Moreover, the results validated that the leached solution was a crystal liquid without solid objects, which is visible to the naked eye, and to rule out the presence of CuO NPs, the authors performed dynamic light scattering (DLS) analysis and did not detect particles. Therefore, the microbiological and DLS results confirmed that the antibacterial activity was mainly due to Cu^+2^ ions. In relation to this research, we could highlight the microbiological analysis that they made to the leached particles by the plate counting method to confirm that the death of bacteria is effectively caused by copper ions from the slight dissolution of CuO in water.

Another mechanism of antibacterial activity is the direct contact of CuO NPs with bacteria. In this case, the CuO NPs present in functionalized materials could adhere to the surface of the bacteria by electrostatic forces and molecular interactions, and the NPs smaller than 10 nm in diameter could pass through the bacterial cell wall [40,61]. Upon reaching the cytoplasmic membrane, CuO NPs could enter the cytoplasm by endocytosis and direct diffusion [40,92] (see Figure 5(b)). According to Cossart and Helenius [93], endocytosis is a process where fluid, solutes, ligands, particles, and other extracellular contents are enveloped with the cytoplasmic membrane to form vesicles that remain inside the cell. On the basis of this definition, CuO NPs could be encompassed by the cytoplasmic membrane and form a vesicle that subsequently fuses with internal components in the cytoplasm. Conversely, the way of introduction to the cytoplasm by the direct diffusion of CuO NPs can be related to the adherence of these nanoparticles to the cytoplasmic membrane, and simultaneously with the generation of ROS on its surface that causes an increase in cell permeability, which leads to the uncontrolled transport of nanoparticles into the cell through this membrane [94]. Direct diffusion could also be due to the preference of CuO NPs toward the lipid head group or the tail (phospholipids) component of the cytoplasmic membrane, and this is related to their hydrophobic or hydrophilic nature [40]. Finally, nanoparticles being in the cytoplasm can bind to DNA molecules causing the helical structure disorganization, the inhibition of DNA replication, protein denaturation, and consequently cell death [92,94]. Apart from that and in parallel to this mechanism, CuO NPs could release ions and trigger the effects associated with them, which were previously discussed.

Among the mechanisms of the antimicrobial activity of CuO NPs present in the textile material and that are in contact with the bacteria, there are ROS, as shown in Figure 5(c). ROS are a type of molecules that contain oxygen and represent the most important group of oxidants that includes the hydroxyl radical (OH^●^), superoxide ion (O_2_^●−^), hydrogen peroxide (H_2_O_2_), and organic peroxides, among others [95,96]. CuO NPs and Cu^+2^ ions induce the generation of ROS [40,62,87,97]. Moreover, it should be considered that, at a nanometer scale, there is a formation of more ROS per unit weight, and due to its excess production and disruption to the bacterial defense mechanisms, programmed cell death is activated, and triggers oxidative stress [40,94]. According to Bedlovicova et al. oxidative stress is defined as a state when the equilibrium between the antioxidative defense of cell and oxidants is disrupted by the effect of excess of the oxidants such as ROS. Hence, this oxidative stress has a higher lethality than the initial ROS caused by CuO [40]. In addition to the aforementioned, excess ROS can lead to lipid peroxidation, the oxidation of proteins, and damage to DNA and RNA, and cause bacterial death [67,87,98].

## 4. Wash Durability Test and Uses of Textiles Functionalized with CuO Nanoparticles

A practical way to evaluate the durability of the antimicrobial properties of textiles functionalized with CuO NPs is through washing cycles. Table 1 and Table 2 exhibit the number of washing cycles and the conditions and methodologies employed in the washing tests of the different papers studied. The washes can be conducted by using simple protocols, such as shaking a cotton fabric in distilled water at room temperature for a certain time and then drying it [54], or by following the methodologies of international technical standards, such as AATCC 61 (2A), which comprises washing textile samples in stainless steel beakers containing 50 stainless steel balls and a 150-mL wash solution. This solution is prepared by dissolving a standard reference detergent without an optical brightener in distilled water. The beakers are placed inside a laundering machine pre-heated at 49 °C, which will be kept at a constant rotation of 40 rpm for 45 min. At the end of the laundering process, the textile samples are rinsed thrice with distilled water at 40 °C for 1 min and dried at a temperature not exceeding 71 °C [62,65,99].

The results reported in the literature verify that washing cycles produce a slight release of CuO NPs from a cotton textile functionalized with these same nanoparticles. Notwithstanding, the textile has the ability to resist its antibacterial activity against Gram-positive and Gram-negative bacteria even after a minimum of 10 washing cycles. However, the percentage of bacterial reduction decreases as the number of washing cycles increases [54]. Another study has affirmed that the antibacterial activity of fabrics functionalized with CuO NPs after 30 washes is slightly lower than that without washing and that this behavior is associated with a decrease in the amount of CuO NPs on the fabric surface due to the dissolution of copper ions in the washing solution [52,63]. Thus, repeated washings do not significantly affect the antibacterial behavior of fabrics functionalized with CuO NPs, thereby indicating the durability of the antimicrobial property [56,63,67,76]. The presence of CuO NPs may, nonetheless, slightly decrease the wettability of treated cotton fabrics [57]. These results corroborate that this type of fabric, which is still under study at the laboratory level, could be utilized as bed materials in hospitals and for the manufacture of uniforms for health-care workers and for patient clothes, such as gowns [53,59,60].

## 5. Cytotoxicity of Textiles Functionalized with CuO Nanoparticles

The cytotoxicological study of textiles functionalized with CuO NPs is still under investigation, which is why there is little information in this regard. Sight et al. [100] functionalized fabrics with CuO NPs via in situ and ex situ using an ultrasound method. The fabrics obtained were intended for use as laboratory coats, scrubs, uniforms, curtains, patient apparels, and bed linens in health-care settings. Therefore, CuO NPs present in the fabrics would have minimal direct contact with human skin cells, hence, they preferred to evaluate cytotoxicity by an indirect method, that is, through the leaching of CuO NPs from the fabrics and, in practice, they could be produced by the dissolution of nanoparticles in the body fluids from the skin. Another reason is that some investigations have claimed that dissolution is the main agent in the toxicity of nanoparticles and involves ions and particulate species that trigger ROS production and damage to the DNA of cells [92,101]. The authors evaluated the cytotoxicity of fabrics functionalized with CuO NPs employing the microculture tetrazolium (MTT) assay, which is a standard colorimetric method for measuring the cell viability. The cells analyzed were human dermal fibroblasts (HDF) and, considering the risk of nanoparticles entering the human body through diverse routes and their possible interaction with sensitive organs, the authors also employed human hepatocellular carcinoma cells (HepG2). The leached solution, obtained after contacting the fabric with a cell culture medium for a certain time, was exposed to HDF and HepG2 cells to detect its potential toxicity. The results validated that leached CuO NPs did not induce toxicity in HDF cells, but there was slight cytotoxicity in HepG2 cells. Finally, the reading confirmed that the nanoparticles did not cross the skin [102] and that the fabrics functionalized with CuO NPs were not toxic to human skin cells.

## 6. Conclusions and Future Perspectives

Functional textiles with CuO NPs are obtained through two methods, namely, in situ and ex situ, and these methods allow textiles to acquire outstanding antimicrobial properties, which are highly dependent on the size and shape of nanoparticles. The mechanism of antimicrobial activity of textiles functionalized with CuO NPs starts with the adsorption of bacteria on the textile surface. From there, three possible mechanisms were reported in the literature: the release of copper ions, the direct contact of CuO NPs with bacteria, and the production of reactive oxygen species. Copper ions coming from the slight dissolution of CuO NPs and the nanoparticles themselves, both present in the functionalized textile, are electrostatically bonded with the bacterial cell wall. Ions or NPs then cross the cell wall and come into contact with the cytoplasmic membrane, which can be penetrated through different pathways. Once inside the cytoplasm, the ions or NPs cause the death of bacteria. Therefore, textiles functionalized with CuO NPs have antibacterial properties against Gram-positive and Gram-negative strains, have durability after several washing cycles, and are not toxic to human skin cells. The use of this type of fabric in health facilities, that is, as uniforms for doctors, nurses, cleaning personnel and patients, could contribute to the reduction of HAIs.

Moreover, in the future, textiles with antimicrobial and antiviral properties that resist the washing process will be important. This could greatly reduce the number of pathogens transferred from person to person. Copper is cheaper compared with silver and is easily available; therefore, cotton fabrics functionalized with copper oxide nanoparticles are cost-effective. Copper oxide nanoparticles have the capacity to inactivate various viruses, such as rhinovirus 2, yellow fever, influenza A, measles, parainfluenza 3, Punta Toro, human immunodeficiency, adenovirus type 1, cytomegalovirus, vaccinia, human influenza A, hepatitis C, and herpes simplex type 1 [34,103,104,105,106]. Recently, no viable SARS-CoV-2 has been measured after 4 h after application on copper surfaces [107]. Moreover, a coating that comprises cuprous oxide (Cu_2_O) particles bound with polyurethane onto glass or stainless steel inactivates the SARS-CoV-2 very quickly, and the viable viral counts drop by an average of approximately 99.9% in 1 h [108].

Therefore, a functionalized textile with CuO could have antiviral properties. An antiviral performance test for textile products is crucial, and this could be tested with ISO 18,184 [109,110]. Functionalizing textiles with CuO is essential, and obtaining textiles can inactivate SRAS-CoV-2.

## Figures and Tables

**Table 1:** Summary of the functionalization of textile materials with CuO NPs conducted by the in situ method.

Precursor	Textile Substrate	Shape of Particles/Structure	Crystal Structure/Size of Particles	Application Method	Additional Treatment/Others	Functionality	Washing Testing	Antimicrobial Activity	Antimicrobial Test	Ref.
0.005 M Cu(Ac)_2_·XH_2_O	Cotton	NR	15 nm	Sonochemical/ultrasonic irradiation (20 kHz)	No additional treatment	Antibacterial activity	NR	*E. coli* (ATCC 10407), *S. aureus* (ATCC 29067)	NR	[42]
0.04 mol Cu(CH_3_COO)_2_·H_2_O	Cotton	NR	~10–20 nm	Sonochemical/ultrasonic irradiation (20–27 kHz)	No additional treatment	Antibacterial activity	NR	*E. coli* (ATCC 10407)	USP 51	[43]
4.8 × 10^−4^ M CuSO_4_·5H_2_O	Cotton	NR	10 nm	Ultrasonic irradiation (20 kHz)	Fabric was washed with 5% sodium dodecyl sulfate at 40 °C for 1 h.	Antibacterial activity	NR	*S. aureus*, *E. coli*	NR	[44]
8 g Cu(CH_3_COO)_2_·H_2_O	Cotton	NR	60–80 nm	Sonochemical	Roll to roll coating	Antibacterial activity	65 cycles The hospital protocols of hygienic washing (75 °C) was used. EN ISO 6330	*S. aureus* (ATCC 6538), *E. coli* (ATCC 8739)	BS EN ISO 20743:2007	[52]
0.01 M Cu(Ac)_2_	Cotton	NR	Monoclinic Crystallite size: ~10 nm. Particle size on cotton: ~30 nm	Sonochemical (20 kHz)	Roll-to-roll coating	Antibacterial activity	NR	*S. aureus*, *Acinetobacter baumannii* (*A. baumannii*), *E. coli*	NR	[53]
0.01 M Cu(Ac)_2_·XH_2_O	Cotton and hybrid fabric polyester: cotton (65:35)	NR	~80 nm	Sonochemical installation	Roll to roll coating	Antibacterial activity	NR	*S. aureus*, *E. coli*	NR	[45]
0.005 mol CuSO_4_·5H_2_O	Cotton	NR	NR	Ultrasound irradiation (20 kHz)	0.001 M sodium dodecyl sulfate, cetyl tri-methyl ammonium bromide, triton X-100, and 0.001 M alkyl hydroxy-ethyl dimethyl ammonium chloride surfactants were used in the functionalization process.	Antibacterial activity	5 and 10 cycles at room temperature	*E. coli*, *S. aureus*, *Candida albicans* (*C. albicans*), *Microsporum canis* (*M. canis*)	AATCC 100 (2004)	[54]
Cu(Ac)2·XH2O	Cotton	NR	NR	Sonochemical/ultrasonic irradiation (20 kHz)	Two pilot scale machines	Antibacterial activity	NR	*S. aureus* (ATCC 6538), *Pseudomonas aeruginosa* (*P. aeruginosa*) (ATCC 15442), *A. baumannii* (NCTC 10303), *E. coli* (ATCC 8739), *methicillin-resistant Staphylococcus aureus* (MRSA) (NCTC 10442)	BS EN ISO 20743:2007	[46]
Copper ions	Cotton	NR	Monoclinic Various sizes	Ultrasonic irradiation (19 kHz)	No additional treatment	Antibacterial activity	65 cycles Washed at 75 °C	*S. aureus*, MRSA, *A. baumannii, E. coli, P. aeruginosa*	NR	[55]
Cu(NO_3_)_2_·3H_2_O	Polyaniline/cotton	NR	Monoclinic	Ultrasonic treatment (40 kHz)	Aniline polymerization	Antibacterial activity, electrical conductivity	NR	*S. aureus*, *E. coli*, *C. albicans*	NR	[47]
0.1–15% owf CuSO_4_·5H_2_O	Cotton	Rod and wheat-like	40–70 nm	Dip coating	Anti-creasing agent in the functionalization process	Antibacterial activity	10 and 30 cycles. 1 mL/L standard detergent at 60 °C-30 min was used.	*S. aureus*, *E. coli*	AATCC 100 (2004)	[56]
0.2 M CuSO_4_	Cotton	NR	NR	Dip coating	The fabric was cleaned with hydrogen peroxide, ammonium hydroxide. 3-chloropropyltriethoxisilane, diethanolamine in the functionalization process	Catalytic activity, antibacterial activity	NR	*S. epidermidis*, *E. coli*	Diffusivity zone inhibitory tests	[49]
0.2 M Cu(CH_3_COO)_2_	Cotton	NR	Crystallite size on cotton: 10.6 nm	Dip coating	The fabric was washed with a 1-g/L nonionic detergent solution as a pretreatment.	Photocatalytic activity, antibacterial activity	5 cycles	*S. epidermidis*, *E. coli*	AATCC 100	[57]
10 mM CuSO_4_	Cotton	NR	NR	Dip coating	The fabric was washed with nonionic agent Felosan RG-N and modified with 20 mL of the oxalic acid solutions of different concentrations (4, 6, and 10 *w/v%*).	Antibacterial activity	NR	*E. coli* (ATCC 25922, NCTC 13846, ATCC BAA-2469), *S. aureus* (ATCC 25,923 and 43,300), *P. aeruginosa* (ATCC 27853), *C. albicans* (ATCC 24433), *K. pneumoniae* (ATCC BAA-2146)	ASTM E 2149-01	[48]
0.2 M Cu(CH_3_COO)_2_	Polyester	Cauliflower-like	Crystallite size on PES: ~27.5 nm. Particle size on PES: 222.5 nm	Dip coating	The fabric was washed with nonionic detergent as a pretreatment. The *Seidlitzia Rosmarinus* plant *(Keliab)* solution was used as reagent alkaline in the functionalization process.	Photocatalytic activity, self-cleaning properties, UV protection, antibacterial activity	NR	*S. aureus*, *E. coli*	AATCC 100	[58]
Cu(CH_3_COO)_2_	Wool	NR	Crystallite size on wool: ~8.5 nm. Particle size on wool: 36.5 nm	Dip coating	The fabric was washed with nonionic detergent as a pretreatment. The *Seidlitzia Rosmarinus* plant *(Keliab)* solution was used as reagent alkaline in the functionalization process.	Antibacterial activity, UV protection	1 and 5 cycles	*S. aureus*, *E. coli*	AATCC 100	[50]
5, 25, 125, and 250 mM % CuSO_4_·5H_2_O	Cotton	Spherical	40–100 nm	Dip coating + shaking	*Cassia alata* leaf extract as a pretreatment	Antibacterial activity	5, 10, and 15 cycles	*E. coli*	Diffusion method	[59]
2 mmol Cu(Ac)_2_·2H_2_O	Cotton	Round clusters	Monoclinic 40–94 nm	Ultrasonic-mediated dip coating	Two metal oxide sols were prepared: TiO_2_ and CuO sol syntheses. The modification of the cotton surface was achieved by the incorporation of citric acid (CA) and polyethylene glycol (PEG) to improve the attachment. The amount of TiO_2_ and CuO on cotton fibers was in the range of 10–12 *wt%*.	Antibacterial activity	10 cycles The treated cotton was washed with 2 g/L nonionic detergent at 50 °C-15 min, rinsed with water, and dries at 100 °C-5 min.	*S. aureus* (ATCC 6538), *E. coli* (ATCC 25922)	Agar diffusion test Dynamic shake test.	[60]
10 mM CuSO_4_·5H_2_O	Cotton	NR	NR	Dip coating + dry + cure	Fabric was washed with nonionic agent Felosan RG-N. Succinic, 1,2,3,4-butanetetracarboxylic and citric acids in the functionalization process.	Antibacterial activity	NR	*S. aureus* (ATCC 25923), *E. coli* (ATCC 25922)	ASTM E 2149-01	[61]
18 *w/w%* CuSO_4_·5H_2_O	Cotton	Various morphologies	Monoclinic Crystallite size on cotton: ~15.9 and 12.4 nm	Dip coating + shaking	The fabric was washed with 2 g/L nonionic detergent at 60 °C for 45 min as a pretreatment.	Antibacterial activity	10, 20 and 30 cycles AATCC 61(2A)-1996	*E. coli*, *S. aureus*	ISO 20645:2004 and AATCC 100:2004	[62]
4 *wt%* CuSO_4_	Cotton	NR	Various sizes	Dip coating + shaking	The fabric was washed with 1 g/L nonionic detergent at 60 °C for 20 min. The citric acid and sodium hypophosphite were used as a stabilizing protective and reducing agent, respectively, in the functionalization process.	Antibacterial activity	10, 20 and 30 cycles AATCC 61(2A)-1996	*E. coli*, *S. aureus*	AATCC 147 and AATCC 100:2004	[63]
0.022 g MCu(CH_3_COO)_2_	Cotton	Needles, prolate spheroidal	L: 80 nm, D: 10 nm	Coating/dyeing process + ultrasonic irradiation (20 kHz)	The 0.16 g reactive orange 16 (RO16) and 0.16 g reactive black 5 (RB5) dyes were used in the functionalization process	Antibacterial activity	Not specified	*E. coli*	NR	[64]
1 mm CuCl_2_	Cotton	Nanosphere	25–30 nm	Dip padding + microwave irradiation (2455 MHz)	The fabrics were treated with 10% (*v/v*) mercaptoacetic acid in the presence of *para*-toluene sulphonic acid.	Antibacterial activity, UV protection, flame retardation	10, 20, and 30 cycles AATCC 61(2A)-1996	*S. aureus*, *E. coli*	NR	[65]
0.5 M Cu(NO_3_)_2_·3H_2_O	Paper (cellulose)	Nano-leaves	W: 300 nm, L: 700 nm, T: 30 nm	Soak + drying	NR	Antibacterial activity	NR	*E. coli* (ATCC 8739), *S. aureus* (ATCC 6538)	NR	[66]
0.2–30.0% owf Cu(CH_3_COO)_2_·H_2_O	Cotton	NR	NR	Exhaust dyeing	NR	Antibacterial activity, UV protection	20 cycles ISO 105-C06:1994	*E. coli* (ATCC 25922)	ASTM E 2149-01	[51]
Copper wires—1 mm diameter	Cotton	NR	<100 nm	Spark discharge (1 kHz)	Ultrasonic pulse generator to avoid NP agglomeration in the functionalization process	Antibacterial activity	15 cycles	*S. aureus*, *E. coli*	AATCC 100	[67]
Copper cathodic	Polyester, polyamide	NR	NR	Cathodic cage plasma deposition	The argon atmosphere was used in the process.	Antibacterial activity	NR	NR	NR	[68]

NR = non-reported; L = length; D = diameter; W = width; T = thickness; owf = on-weight-fabric.

**Table 2:** Summary of the functionalization of textile materials with CuO NPs by the ex situ method.

Precursor	Textile Substrate	Shape of Particles/Structure	Crystal Structure/Size of Particles	Application Method	Additional Treatment/Others	Functionality	Washing Testing	Antimicrobial Activity	Antimicrobial Test	Ref.
10 mM Cu(CH_3_COO)_2_·H_2_O	Woven	Caterpillar-like Ellipsoidal body shaped architectures:	Monoclinic W: ~80–100 nm, L: 140–160 nm	Drop casting	CuO nanostructures were synthesized by green wet-chemical method	Energy storage	NR	–	–	[71]
CuO NPs, throwing stones mode	Cotton	Ellipsoidal	200–400 nm	Sonochemical/Ultrasonic irradiation (19 kHz)	No additional treatment	Antibacterial activity	NR	*E. coli*, *Klebsiella pneumoniae* (*K. pneumoniae*), *S. aureus*, MRSA	BS EN ISO 20743:2007	[72]
0.75–1.4 *wt%* CuO NPs	Cotton	NR	NR	Sonochemical	No additional treatment	Antibacterial activity	65 cycles Washed at 75 °C	*S. aureus*, *E. coli*	NR	[73]
1 M CuSO_4_	Cotton	NR	NR	Simple dipping technique	The CuO NPs were synthesized with a copper sulphate solution and *Sida acuta* extract.	Antibacterial activity	NR	*S. aureus*, *E. coli*, *Proteus vulgaris* (*P. vulgaris*)	Agar plates	[74]
CuSO_4_·7H_2_O:H_2_O (1:30)	Bamboo rayon	NR	NR	Dip coating	Bamboo rayon fabric grafted with acrylic acid	Antibacterial activity	50 cycles	*S. aureus*, *E. coli*	AATCC 100 (2004)	[75]
CuSO_4_	Cotton	NR	NR	Dip coating	The CuO NPs were synthesized with a copper sulphate solution and 50 mL of *Ruellia tuberosa* aqueous extract.	Antibacterial activity	NR	*E. coli*, *K. pneumoniace*, *S. aureus*	Agar plates	[70]
3.2; 8.0; 16 g/m^2^ CuO NPs	Cotton	NR	<50 nm	Dip coating + shaking	Tetrahydrofuran, polydimethylsiloxane, and silanes were used in the process.	Superhydrophobicity, antibacterial activity	Triton-X, non-ionic detergent (1 g/L) was used to wash.	*E. coli* (ATCC 25922), *S. aureus* (ATCC 25923)	AATCC 100 (2004)	[76]
1 mg CuO NPs–woven fabric 14 mg CuO NPs–non-woven fabric	Cotton	NR	Monoclinic 50 nm	Immersion	The fabric was washed with hydrochloric acid. The chemical precipitation method to synthesize CuO NPs from 0.2 M Cu(NO_3_)_2_·2H_2_O was used.	Antibacterial activity	NR	*S. aureus*, *Bacilos subtilis (B. subtilis*), *E. coli*, *P. aeruginosa*	Agar diffusion	[69]
0.1 M CuSO_4_	Cotton	NR	50 nm	Exhaustion + pad–dry–cure	The CuO NPs were synthesized by a wet-type chemical method. Microencapsulation was conducted by the ionic gelation method using 3% sodium alginate and 2% CuO NPs.	Antibacterial activity	5 and 10 cycles AATCC61 (1A)-2001	*S. aureus* (ATCC 6538), *E. coli* (ATCC 11,230 and 8739)	AATCC 147 and AATCC 100	[77]
0.3 and 1.0%(*w/w*) CuO	Polypropile-ne	Round clusters	<0.5 µm	Spinning process	The fibers were produced with polypropylene chips and CuO microparticles. Melt was performed using a twin screw extruder.	Antibacterial activity	NR	*E. coli* (ATCC 25922)	JIS L 1902:2002	[78]

NR = non reported; L: length, W: width.
